# Does Anyone Know the Answer to that Question? Individual Differences in Judging Answerability

**DOI:** 10.3389/fpsyg.2015.02060

**Published:** 2016-01-13

**Authors:** Bodil S. A. Karlsson, Carl Martin Allwood, Sandra Buratti

**Affiliations:** Department of Psychology, University of GothenburgGothenburg, Sweden

**Keywords:** question answerability, judgments, consensus, epistemic beliefs, epistemic preference, optimism

## Abstract

Occasionally people may attempt to judge whether a question can be answered today, or if not, if it can be answered in the future. For example, a person may consider whether enough is known about the dangers of living close to a nuclear plant, or to a major electricity cable, for them to be willing to do so, and state-authorities may consider whether questions about the dangers of new technologies have been answered, or in a reasonable future can be, for them to be willing to invest money in research aiming develop such technologies. A total of 476 participants, for each of 22 knowledge questions, either judged whether it was answerable today (current answerability), or judged when it could be answered (future answerability). The knowledge questions varied with respect to the expected consensus concerning their answerability: *consensus* questions (high expected consensus), *non-consensus* questions (lower expected consensus), and *illusion* questions (formulated to appear answerable, but with crucial information absent). The questions’ judged answerability level on the two scales was highly correlated. For both scales, consensus questions were rated more answerable than the non-consensus questions, with illusion questions falling in-between. The result for the illusion questions indicates that a feeling of answerability can be created even when it is unlikely that somebody can come up with an answer. The results also showed that individual difference variables influenced the answerability judgments. Higher levels of belief in certainty of knowledge, mankind’s knowledge, and mankind’s efficacy were related to judging the non-consensus questions as more answerable. Participants rating the illusion questions as answerable rated the other answerability questions as more, or equally, answerable compared to the other participants and showed tendencies to prefer a combination of more epistemic default processing and less intellectual processing.

## Introduction

In daily life and in science people are sometimes faced with questions that they do not immediately know the answer to. Examples are questions such as “Is it safe to use a cell-phone daily?”, “Can this person be considered guilty beyond a reasonable doubt?”, or “Does this medicine have serious side effects?” Moreover, if a person thinks that a question currently has not been answered, they may consider how long it will take before it will be answered, or if it can be answered at all. For other questions, people may think they know the answer, only to find out that their judgment was premature. Many tragic human catastrophes may, at least in part, have occurred because people misjudged the answerability of important questions, e.g., the Thalidomide catastrophe in the 1950s.

The notion that questions can have correct answers is commonly taken for granted in everyday life and in science. In the present research, we use the term *answer* to mean an answer that is correct and that is provided with good arguments at a relevant level of specification. In this context there are thorny philosophical issues related to, for example, the concepts of *truth* and *knowledge* that have been debated by philosophers for centuries. However, this paper does not try to solve such issues. Thus, for example, at a philosophical level, it may presently not be possible to convincingly claim that there is an absolute difference between answerable and non-answerable questions. In spite of this, we note that people, both in science and in everyday life in fact make judgments about when in the future, questions judged not to have been answered, are likely to be so, if ever. The present study investigated people’s subjective judgments of questions’ answerability.

Such judgments are important because they may influence other judgments and decisions relevant in everyday life, for example decisions whether to allow new techniques or substances in specific contexts such as in medicines, clothes, foods, or building materials. An example of a future answerability judgment is how many test trials should be made before we can be sure that a new drug is not dangerous to the public? Other contexts where answerability judgments are made in everyday life, including work life, concern allocation of resources to research project applications or to public and other inquiries. For example, if it is reasonable to think that a research project can answer its questions during the time the research money is applied for? As far as we know no research has studied answerability judgments in the broad sense discussed here.

Much of previous research on question answerability judgments has been limited to questions where people tend to agree on the answer and has tended to study isolated aspects of the broader answerability question, for example, if I myself or someone else knows the answer to a posed question. Thus, studies of “I know/don’t know” judgments have been made in different fields of psychology. To illustrate, research in forensic psychology has explored participants’ ability to separate out questions on “information not seen” in a video clip (e.g., Candel et al., 2007; Roebers et al., 2007; Scoboria et al., 2008; Frey and Scoboria, 2012; Buratti et al., 2014), in general finding a lack in this ability. In this context, Frey and Scoboria (2012) suggested a certain mental ability called “skill” that denotes the ability to separate an answerable from a non-answerable question with respect to information (not) seen. In experimental memory research “Don’t know” judgments have been studied for various types of simple semantic and episodic information (e.g., Kolers and Palef, 1976; Glucksberg and McCloskey, 1981; Hampton et al., 2012). Glucksberg and McCloskey (1981) concluded that when people attempt to answer a question, a memory search is first made to identify facts that may be relevant to the question, and if found, then such facts are further considered in detail in order to assess if they can be used to answer the question. In a study with several experiments, Hampton et al. (2012) tested if people can use awareness of their ignorance to deliver improved test consistency over time. Their results showed that there is certain factual knowledge that the participants saw as “known unknowns”, that is, facts that they consistently knew that they did not know. When the participants answered general knowledge questions, there existed a high consistency between test and retest in answers if they were allowed to answer “I don’t know” in addition to “TRUE” and “FALSE”. Thus, participants gave stable unsure responses to certain items. However, these results were not found for other types of knowledge, for example, autobiographical memories, beliefs and aspiration.

Research on people’s confidence in their answers to different types of memory questions has in many contexts found a tendency for people to show overconfidence in their responses (see review by Griffin and Brenner, 2004). Overconfidence is especially common when the issue considered is less well known to the person. Research on metacognition in psychology has also investigated judgments of *others’* knowledge. To illustrate, Johansson and Allwood (2007) found that students were more confident in other students’ general almanac knowledge than in their own. However, although the level and accuracy of people’s confidence may influence the processes generating answerability judgments, no confidence judgments were collected in the present research and for many of the questions used in the present research there is no general consensus on their correct answer.

Beliefs about differences in knowledge between individuals and groups (e.g., experts and laypeople) have been addressed in educational psychology orientated toward science communication (e.g., Hofer and Pintrich, 2002; Scharrer et al., 2013, 2014; Shtulman, 2013) and in risk research (e.g., Kahan et al., 2011). This line of research shows that individual differences may contribute to how people experience the credibility of knowledge and knowing in general, in different domains. For example, Shtulman (2013, p. 207) studied students’ epistemic attitudes to two types of belief: scientific and supernatural. The results showed that the participants’ confidence in both types of beliefs was more clearly related to their experienced social consensus about the belief and less related to their ability to provide justifications for their beliefs. Furthermore, also relevant to the present research the most common form of justification was “deference to the opinions and conclusions of others”.

Evidence in risk research shows that differences in cultural values such as hierarchical/egalitarian and individualistic/communitarian may shape individuals’ beliefs about the existence of scientific consensus on such topics as climate change and disposal of nuclear waste (Kahan et al., 2011). Other research in this area has indicated that for some issues people believe that there are clear limits to what experts and science can know (Sjöberg, 2001). This type of differences in the approach to knowledge and science in general may well affect question answerability judgments.

Conceptions about ignorance are also likely to be important when people make answerability judgments. For example, if people think that very little is known in an area, they may think that questions relating to the area are less likely to be answered. Risk researchers (e.g., Ravetz, 1987, 1993; Faber et al., 1992), sociologists (e.g., Smithson, 1993; Gross, 2007; Croissant, 2014), computer scientists (e.g., Armour, 2000) and philosophers (e.g., Rescher, 2009; van Woudenberg, 2009) have discussed *ignorance*, taken in a broad sense. Researchers have also presented taxonomies of ignorance (e.g., Faber et al., 1992; Smithson, 1993; Armour, 2000; Gross, 2007; Rescher, 2009; Croissant, 2014). For example the taxonomy of Armour (2000) highlights that ignorance can be more or less deep. Faber et al.’s (1992) taxonomy is also relevant in the present context since it separates communal ignorance (the ignorance of a group) from individual ignorance (the ignorance of a person). The question answerability judgments examined in the present study potentially include deliberations about the ignorance of other people or groups, including the whole of mankind. As far as we know, such question answerability judgments have not been thoroughly studied before. The present study contributes by studying broad answerability judgments of questions about factual states of the world where the answer may not be generally agreed upon or simply is unknown.

Answerability judgments can be classified in different ways. For example, they can be seen as a divergent thinking task because the person facing an answerability judgment may consider several alternative interpretations of, or answers to, the question that is judged (see e.g., Gilhooly et al., 2007, on divergent thinking tasks). For answerability judgments of questions, people may first consider if they have heard the specific question before, and if they or someone else may know the answer. If so, the question may be judged to be answerable. Later they may make other more general considerations, for example with respect to how much is known in the area the question belongs to, if there are alternative meanings to the question, or if general ways that the question could be answered can be thought of. They may also consider if it is likely that the answer to the question will ever be found, and if so, how long time it will take, for example based on the amount of work required to answer it. Each of these aspects may lead to further deliberations.

A common conclusion is that people have a tendency to trust socially prevalent understanding (e.g., Perkins, 1993; Cole, 1996; Atran et al., 2005). Socially prevalent understanding will henceforth be called *consensus knowledge* and consensus is seen as a matter of degree. In general, it seems reasonable that people may often seek guidance from what they conceive of as common opinions and attitudes in their environment when making answerability judgments. This guidance may take place both with respect to the answer to the question judged (answer consensus) and with respect to question answerability (answerability consensus). There are at least three types of possible answerability consensus: whether there is *some* answer to the question *today*, whether it can be answered *in the future*, and about the answerability of specific types of questions. Koriat (2008, 2012) showed elegantly that main trends in socially prevalent understanding are an important influence on individuals. To illustrate, Koriat (2008) found high confidence both when individuals in a two-alternative general knowledge task selected the commonly believed answer alternative that was also the correct answer, and when individuals selected the commonly believed, but incorrect, answer.

In this research we investigated answerability judgments of three types of questions: questions for which we expected a high degree of consensus regarding their answerability (consensus questions), questions for which we expected a low degree of such answerability consensus (non-consensus questions), and questions that may appear answerable but where some information necessary to answer it was missing (illusion questions). However, we do not claim that there is an absolute qualitative difference between these question types, rather the difference may be a question of degree. Consensus and non-consensus questions were used in this research because it is of interest to study the consistency in people’s answerability judgments of questions with different degrees of expected answerability. In addition, we were also interested in studying the degree to which individual difference variables influenced questions with different levels of expected consensus about their answerability (further elaborated below). The illusion questions were included in order to examine the extent to which missing information in the questions might be compensated for by the use of other types of information such as conceptions about the answerability of specific types of questions.

We expected that consensus questions would be rated higher in answerability than non-consensus questions (Hypothesis 1). This was partly because we expected that it would be relatively easy for the participants to either provide what they thought was the correct answer to the question (thus showing that is was answerable), or to imagine some easily performed way to get the answer to the question. The illusion questions belonged to geometry and physics and, since we had purposefully eliminated information necessary to answer them, they show similarity to the questions used in the witness psychology and memory studies reviewed above. Moreover, they were designed to appear fairly elementary and computable and we believed many participants (possibly from their school experience) would think that the answer to such elementary computational questions in general is possible to compute. This is in line with Stahl and Bromme’s (2007) suggestion that individuals have preconceptions about the knowledge in different knowledge domains and that these “domain-specific epistemic beliefs” act as a lens through which an individual makes judgments of knowledge. Due to the low number of illusion questions, these were not statistically compared to the other types of questions in level of answerability. But in general we expected some, but not all, participants to notice that information was missing and for this reason that these questions would be given lower answerability values than the consensus questions.

Answerability judgments can be made on different types of scales and it is of interest to understand the extent to which the level of the answerability judgment varies as an effect of the used scale. Therefore we compared two kinds of answerability scales with respect to their effect on the level of answerability judgments. One scale related to the *current* answerability of the question and the other scale to *when*, if ever, a question can be answered. These two scales were included because it is reasonable to think they are commonly used, explicitly or implicitly, when people judge the answerability of questions in everyday life. In addition, we were interested to compare the answerability levels of the judged questions on the two scales since if the rank order in answerability is stable between the two scales this is an indication of some stability in the processes generating the answerability judgments.

### Individual Differences and Answerability Judgments

Given the potential importance of answerability in everyday life it is also of interest to study if answerability judgments are influenced by factors that, *per se*, may be irrelevant to their realism. Therefore, we also investigated the relation between answerability judgments and measures of individuals’ cognitive and personality properties, henceforth called individual difference variables, that it seems reasonable to believe will be associated with the level of the answerability judgments. In general it would seem that consensus type questions are more likely to be judged by use of quick and fairly automated processes of a System 1 kind (e.g., Kahneman, 2011; for debate see e.g., Evans and Stanovich, 2013; Keren, 2013). The reason is that socially prevalent knowledge is more likely to be encountered more frequently and thus is more likely to be automatized and taken for granted. In contrast, non-consensus questions may, for related reasons, be more likely to be judged by less automated, and more deliberate and elaborated, processing of a System 2 kind. Thus, we believed the individual difference variables we studied would have more influence on the judgments of the non-consensus questions, compared with the consensus questions (Hypothesis 2). These variables include beliefs about epistemological issues (belief in certainty of knowledge, belief in mankind’s knowledge), belief in mankind’s efficacy, preferred processing type and personal optimism, and are discussed next.

#### Belief in Certainty of Knowledge

Personal beliefs about knowledge and knowing in general are referred to as *global epistemic beliefs* and can be separated from *domain specific epistemic beliefs* which concern beliefs about knowledge in specific domains, such as physics or history. Due to the broad span of questions used in the present study we were primarily interested in global aspects of beliefs about epistemic issues.

Development of global beliefs about certainty of knowledge is assumed to go from a naïve state in childhood where knowledge is seen as certain and is distributed by an authority, to a more sophisticated view where certain (sure) answers may be out of human reach or context dependent (Perry, 1970). We expected that the participants would not have strong detailed personal opinions on every question judged and therefore we speculated that their ratings would be influenced by their “general underlying orientation” (Schuman and Presser, 1996, p. 132) about certainty of knowledge (see also Hahn and Oaksford, 2007; Kammerer et al., 2013). Thus, we expected that people who believed more in certainty of knowledge would give higher answerability ratings (Hypothesis 3). In line with Hypothesis 2, this difference was, for this and all the following hypotheses, expected to hold foremost for the non-consensus questions.

#### Mankind’s Knowledge

Beliefs about the extent and usefulness of mankind’s knowledge as such also illustrate belief about epistemic issues. Given that more knowledge might be expected to be associated with greater possibility to answer knowledge questions, we hypothesized that belief in a larger body of mankind’s knowledge would be correlated with higher answerability judgments (Hypothesis 4).

#### Mankind’s Efficacy

A further type of belief concerns beliefs about mankind’s effectiveness to reach, for example, epistemic goals (e.g., the goal of acquiring knowledge), that is, mankind’s efficacy. We expected that participants who believed more in mankind’s efficacy would tend to judge questions as more answerable (Hypothesis 5).

#### Epistemic Preference

Shallow or deep processing might influence answerability judgments. Two kinds of processing preferences are measured by the *EPI-r* scale (Elphinstone et al., 2014): preference for a more automatic default type of processing*, EPI-r Default* (similar to system 1 processing) and preference for a deeper intellectual type of processing measured by *EPI-r Intellectual* (similar to system 2 processing). We expected that participants with default processing preferences would tend to choose interpretations that come quickly to mind and are easy to handle and therefore would find questions more answerable. Furthermore, we expected that preference for intellectual processing would be associated with lower answerability judgments since participants high in this preference would problematize the possible constructions of answers to questions more than participants low in this preference (Hypothesis 6). Our reasoning here was that people with a higher default processing preference may be more prone to rely on general rules of thumb (e.g., preconceptions about a domain or general knowledge beliefs), while intellectual processing may make it easier to notice that crucial information needed to answer the question may be missing.

#### Personal Optimism

Optimism may also affect the level of answerability judgments. People with generalized personal optimism tend to interpret things in a positive way and are less likely to give up (Muhonen and Torkelson, 2005; Carver et al., 2009). As optimists are less likely to give up goals (for example to answer questions) they may think that, given enough attempts, answers to questions will be found. On the other hand, many unsuccessful attempts to find something may lead to a belief that this something does not exist (Hahn and Oaksford, 2007), but maybe less so for optimists. A further part of optimism is that optimists expect good things to happen in uncertain times (Monzani et al., 2014). This could lead questions being seen as more answerable but also to better tolerance of uncertainty in the environment. Optimists may therefore for example be more willing to accept that answers to questions may be uncertain or non-existing. In sum, different features of optimism seem to be theoretically related to answerability in different ways. Therefore we explored optimism in relation to answerability but did not pose a hypothesis in this context.

## Method

### Participants

In total, 476 participants completed the 22 answerability questions in one of the two answerability scale variants (112 men, 313 women, 9 “other”, and 42 “no answer”). The mean age was 28 years (range 18–78 years). Of the 476 participants, 236 (57 men, 165 women, 6 “other”, and 8 “no answer”; mean age 27 years, range 18–78 years) completed the answerability questionnaire using the current answerability scale. The remaining 240 participants (59 men, 154 women, 3 “other”, and 24 “no answer”; mean age 28, range 18–75 years) used the future answerability scale. As a reimbursement, participants participated in a lottery for a cinema ticket.

### Design and Procedure

The present study followed ethical guidelines in Sweden for survey data. Participants were recruited from a pool of adults that had already actively volunteered and signed up for participation in psychological research and can thus be considered consciously aware of participation in general. They were provided with information about the purpose of the study via e-mail and were told that participation was not mandatory. In the email they were informed that their answers only would be used for research purposes, that they could withdraw at any time. The email also provided relevant contact information. Participants gave their consent by clicking on a survey link. When clicking on the link for participation participants were randomized to four groups. Approximately half of the participants were randomized to each of two scale variants: the current answerability scale and the future answerability scale (described below). In order to control for any ordering effects within each scale variant group the order of the question blocks was altered, constituting two order conditions. In the first order condition (the belief in certainty of knowledge-first condition) the participants answered the belief in certainty of knowledge items first, then the answerability questions, and then three other measures-the LOT-r, EPI-r, and mankind’s efficacy, in a randomized order. In the second order condition (the belief in certainty of knowledge items-last condition) the participants answered the questions in the following order: first the answerability questions, then the three other measures-the LOT-r, EPI-r, and mankind’s efficacy-in a randomized order, and last the belief in certainty of knowledge items. Finally, two questions about mankind’s knowledge and background questions were answered.

In total 1462 individuals from participant pools at the University of Gothenburg were invited to answer a web-questionnaire. It was not possible to go back to previous pages in the questionnaire to change answers. If participants left a question unanswered, they were kindly reminded but not forced, to complete the question. This rendered 639 answers. Out of these 639, 476^1^ participants answered all the 22 answerability questions and data from that set of people were used for further analysis. Thus, the response rate was 33%.

## Materials

### Answerability Questionnaire

A questionnaire with 22 questions was prepared. Each question-item consisted of a question to be judged for answerability. We attempted to include a varied sample of questions from different domains, for example, medicine, Swedish grammar and technology (the questions are further described in **Appendix 1**). However, at the same time we kept the total number of questions reasonably low in order to achieve a good response rate and an even answer quality throughout the questionnaire (Galesic and Bosnjak, 2009).

Three types of questions were used. There were eight *consensus questions*. These were questions for which we expected a high degree of consensus that the question is answerable. An example is *“What is the name of our galaxy?”* There were 12 *non-consensus questions* (questions with less expected consensus about the answerability of the question; for example: “*Are humans causing the green-house effect?”*). The remaining two questions were illusion questions in which a crucial detail necessary to compute the answer was missing. For example, in the question *“How large is the area of an ellipse with a minor axis of 2 cm?”* the length of the major axis was absent. Bottoms et al. (2010) found that tricky questions with misleading details (e.g., the Moses-illusion) were more easily detected if they were frequent in a questionnaire. For this reason we kept the number of illusion questions low in order to avoid that participants would identify this special kind of “trick questions”.

In a pre-study 100 participants from a student pool at the University of Gothenburg rated various aspects of the 22 question-items. Participants estimated the proportion of adult Swedes that would agree that the question is answerable (on a scale from 0% to 100%). On average participants judged that 74% of the Swedes would agree that the consensus questions were answerable, but estimated that only 55% of the Swedish population would agree that the non-consensus questions were answerable. Participants further judged 64% of the Swedes would reckon the illusion questions answerable. The difference between consensus and non-consensus questions was significant *F*(2,19) = 23.45, Bonferroni correction (*p* < 0.01) which supported the researchers’ intuitions. However, the illusion questions did not differ significantly from the consensus (*p* = 0.14) nor the non-consensus questions, (*p* = 0.22).

The questions in the present study were organized in pairs so that questions of different categories were matched with each other. There were two pairs of questions containing one illusion and one consensus questions, six pairs containing one consensus and one non-consensus question, and three pairs containing two non-consensus questions. The just described pairs of answerability questions were presented in an order randomized for each participant.

For both scale variants (the current and future answerability scales), the instructions stressed that the judgment task was not to provide the answer as such to the question but to judge the answerability of the question. Moreover, the participants were instructed that “*answerability means that the question can be answered on a level that is exact and relevant enough. E.g., for the question “how tall is the world’s smallest living creature ever”, the answer “less than a meter” is not exact enough to be counted as correct.*” The instructions are further described in **Appendix 2**.

Each screen in the questionnaire presented two questions for which the participants were to give answerability judgments. On the top of each screen was a reminder formulated as follows: “Reminder: for the question to be judged as answerable it is necessary:” (the next three sentences each appeared on its own line) “That at least one person in the world can answer the question. That the answer can be answered correctly and that good arguments for the answer can be provided. That the question can be answered in a sufficiently exact and relevant way.”

**FIGURE 1:**
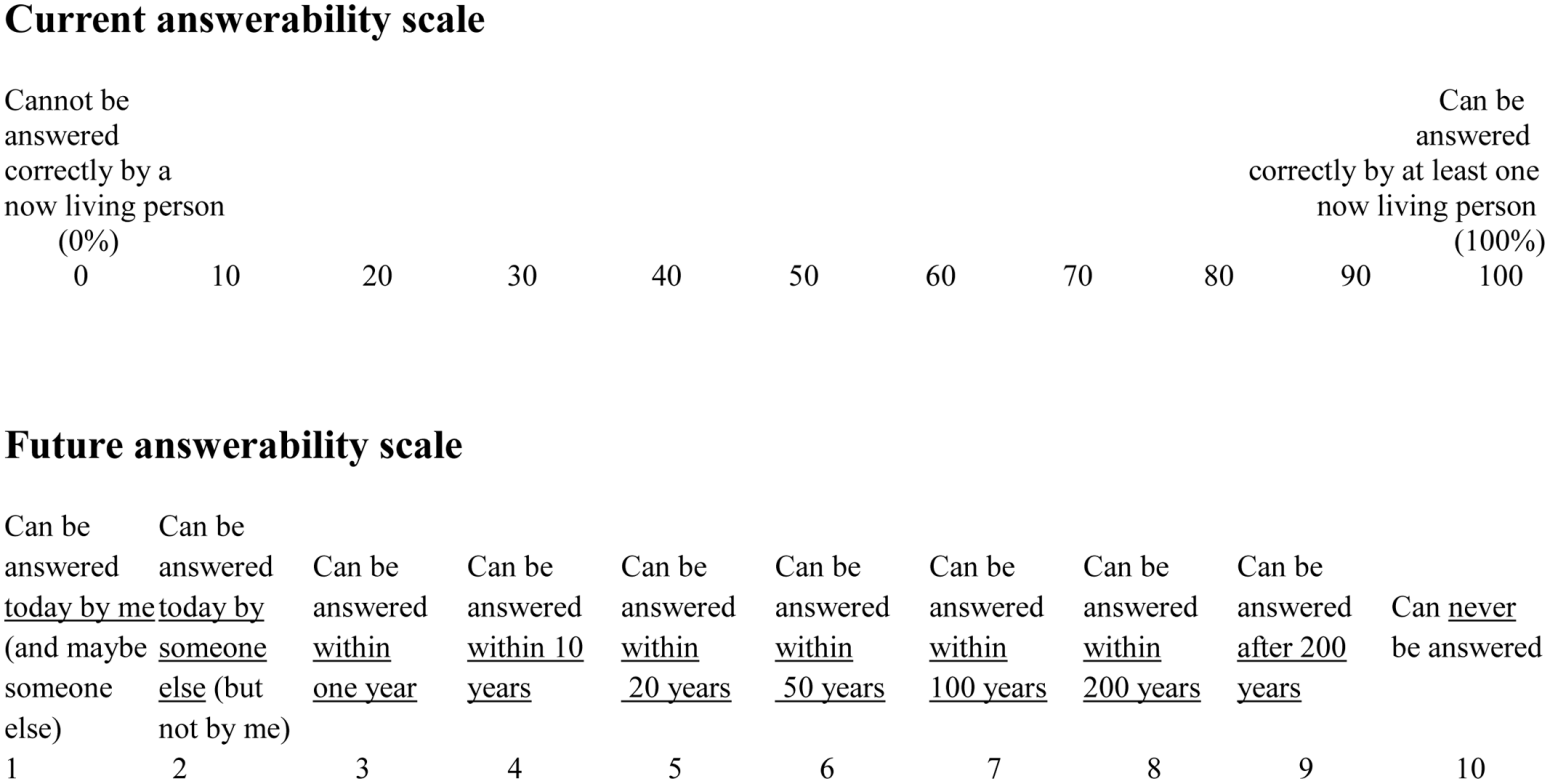
**The current answerability scale and the future answerability scale**.

Two scales were prepared for the answerability judgments. One of the scales, “the current answerability scale” asked the participant to judge the probability that the question could be answered by at least one person now living in the world. The other scale, “the future answerability scale”, more openly directed the judgment to when the question considered can (if ever) be answered. The two scales are shown in **Figure 1.**

### Belief in Certainty of Knowledge

Two items from a factor tapping belief in certainty of knowledge in the epistemic beliefs measure presented by Bråten and Strømsø (2005) were used. Cronbach’s alpha was 0.85. The two items were “*If scientists try hard enough they can find the truth to almost everything*” and “*Scientists can ultimately get to the truth*”. Higher values indicate higher belief in certainty of knowledge. The items were rated on a five-point scale, ranging from 1 = *Do not agree at all* to 5 = *Totally agree*.

### Mankind’s Efficacy

A measure was constructed in order to tap a belief in mankind’s ability to complete tasks and reach goals. The items were similar to self-efficacy items, but instead addressed mankind’s efficacy. The four items were: “*Mankind can always manage to solve difficult problems if it tries hard enough*”, “*Even if hard times threaten mankind, mankind will find ways to reach its goals*”, “*In unexpected situations, mankind will find ways to act*”, and “*Even in unexpected situations I believe mankind can cope well*”. Items were answered on a five-point scale ranging from 1 = *Do not agree*, to 5 = *Totally agree*. Cronbach’s alpha was 0.77.

### Mankind’s Knowledge

Two questions intended to capture beliefs about the extent of mankind’s entire body of knowledge today were asked. The two items were “*How much does mankind know of all there is to know?*” and “*How much does mankind know of all that is important to know?*” Items were answered on a scale ranging from 0 to 100% in intervals of 10. Cronbach’s alpha for the items was 0.66, which although not high can be considered acceptable for research purposes (Streiner, 2010).

### Epistemic Preference (Epi-r)

The Epistemic Preference Indicator-Revised (Elphinstone et al., 2014) measures processing preferences and is an eight-item instrument with two dimensions: default processing (for example, “*When confronting the deep philosophical issues of life I am more inclined to just deal with it, get the job done, and move on*”) and intellectual processing (for example “*In the simplest terms, I have a strong need to study just how and why things happen*”). Questions were answered on a five-point Likert-type scale, ranging from 1 = *Do not agree* to 5 = *Agree completely*. Cronbach’s alpha was 0.71 for default processing, and 0.81 for intellectual processing.

### Life Orientation Test Revised (LOT-r)

Life orientation test revised measures the degree of optimism regarding oneself and has six items (Monzani et al., 2014). An item example is “*In uncertain times, I usually expect the best*”. The items were answered on a five-point scale ranging from 1 = *Do not agree* to 5 = *Totally agree*. Cronbach’s alpha was 0.78.

## Results

Means, medians, SDs, and interquartile range for the answerability judgments for the three types of questions are shown in **Table 1.** Since the future answerability scale starts with two categories of a nominal type, these categories were recoded into the same category in order to make the scale more ordinal. Thus, after recoding 1 = *I or someone else knows the answer to the question*, 2 = *Can be answered within 1 year*, 3 = *Can be answered within 10 years*, etc. Medians and interquartile range were used for the future answerability scale.

**Table 1:** Central tendencies and deviations in the answerability judgments for the current and the future answerability scales.

	Scale			
	
	Current		Future	
		
	Mean	*SD*	Median	IQR
**Question type**				
Consensus	93	12	1.0	0
Non-consensus	47	21	2.5	4.5
Illusion	77	31	1.0	0


*The median refers to median of medians of each question item. IQR = Interquartile range.*

**FIGURE A1:**
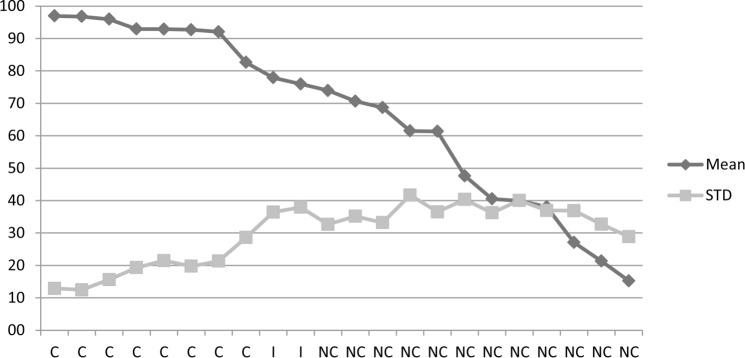
Means and standard deviations for the answerability judgments on the current answerability scale.

As can be seen in **Table 1**, for the current answerability judgments, consensus questions were perceived to be most answerable (*M* = 93%), non-consensus questions the least answerable (*M* = 47%), and illusion questions were rated in between (*M* = 77%). **Figure A1** in **Appendix 3** shows that the answerability questions’ mean ratings ranged from almost 100% answerable to approximately 15% answerable.

For the future answerability judgments consensus questions and illusion questions were rated most answerable (*Mdn* = 1, “*I or someone else knows the answer to the question*”) and the non-consensus questions least (*Mdn* = 2.5, corresponds to “*Can be answered within 1–10 years*”). Most of the respondents (75th percentile) rated the non-consensus questions to be answered within a maximum of 50 years. The response alternative *Can never be answered* was chosen 12% of the time.

Next, analyzes of the differences in the answerability judgments of the consensus and non-consensus items are presented. After this, the analyses of the influence of the individual differences variables on the answerability judgments are described. The illusion questions were excluded from these analyses due to the low number of items in this question category. The analyses of the illusion questions are presented at the end of the result section for each scale type.

### Difference in Level of Current Answerability Judgments

In order to investigate differences between consensus and non-consensus questions a mixed ANCOVA was conducted with the within-subject factor of question type (consensus vs. non-consensus) and, to control for ordering effects, the between-subject factor *order* was used (whether the belief in certainty of knowledge ratings were done *before* or *after* the answerability ratings).^2^ The six individual differences variables were entered as covariates to control for their influence (belief in certainty of knowledge, EPI-default, EPI-intellectual, mankind’s knowledge, mankind’s efficacy, and optimism).

Consensus questions were rated as significantly more answerable than non-consensus questions, *F*(1,208) = 35.41, *p* < 0.001, partial η^2^ = 0.15. We found no effect of the order factor (i.e., no significant difference between if the belief in certainty of knowledge ratings were done before or after the answerability judgments), *F*(1,208) = 0.01, *p* = 0.942. No interaction effect was found between the question type and order factors, *F*(1,208) = 0.25, *p* = 0.618.

### Difference in Level of Future Answerability Judgments

Due to the ordinal properties of the future answerability scale non-parametric tests were used. To estimate the effect sizes the probability of superiority estimator (*PS*) was used, following Grissom and Kim (2012) recommendations. The *PS* estimates the probability that a score randomly drawn from population *a* will be greater than a score randomly drawn from population *b*.

A Mann–Whitney *U*-test showed that there were no differences in answerability judgments for the consensus questions as a consequence of whether the belief in certainty of knowledge items were rated *before or after* the answerability questions (*p* = 0.957). However, there was a significant difference for non-consensus questions due to rating belief in certainty of knowledge before or after the answerability judgments, *U* = 8484.50, *z* = -2.494, *p* = 0.013. *PS* = 0.59. Therefore the results for the future answerability of the non-consensus questions were analyzed jointly and separately per order condition and reported when relevant.

To investigate differences between future answerability for the consensus and non-consensus questions a Wilcoxon signed rank test was used. The consensus questions were rated as being possible to answer in a nearer future than the non-consensus questions, *z* = 10.24, *p* < 0.001, *PS*_dep_ = 0.74.

### Correlations Between Current and Future Answerability Ratings

The median of each question item on the current answerability scale was correlated with the median of each corresponding question item on the future answerability scale. This correlation was significant and very high, *r_s_*(22) = -0.48, *p* < 0.03. Thus, on average, the questions considered unlikely to be answered today were considered to be answered in a more distant future. (The negative sign emerges because the future answerability scale values are higher in a more distant future).

### Individual Difference Variables and Current Answerability

The means and SDs for the individual difference variables and the current answerability ratings of consensus, non-consensus and illusion questions and Pearson correlations between these variables are shown in **Table 2.**

**Table 2:** Current answerability scale ratings: means and SDs and Pearson correlations between the individual difference variables and the answerability ratings of the consensus, non-consensus, and illusion questions.

	*M* (*SD*)	1	2	3	4	5	6	7	8	9
(1) Belief in certainty of knowledge	2.5 (1.2)		0.29^∗∗^	-0.02	0.31^∗∗^	-0.04	0.17^∗∗^	-0.02	0.28^∗∗^	0.03
(2) EPI-default	2.8 (0.91)			-0.23^∗∗^	0.12^∗∗^	0.02	0.20^∗∗^	0.07	0.10	-0.08
(3) EPI-intellectual	3.2 (0.96)				0.12^∗^	-0.07	-0.06	-0.03	-0.09	-0.13
(4) Mankind’s Efficacy	3.3 (0.80)					0.22^∗∗^	0.15^∗∗^	0.08	0.22^∗∗^	-0.05
(5) Mankind’s Knowledge	26 (19)						0.05	-0.04	0.11	0.08
(6) Optimism	3.4 (0.78)							0.03	-0.13	-0.14^∗^
(7) Consensus	93 (12)								0.16^∗∗^	0.21^∗∗^
(8) Non-consensus	47 (21)									0.20^∗∗^
(9) Illusion	77 (31)									


*^∗^*p* < 0.05, ^∗∗^*p* < 0.01.*

#### Consensus and Non-Consensus Questions

There were no significant correlations between the individual differences variables and the answerability ratings on the current answerability scale for the consensus questions. However for the non-consensus questions, people that rated a high belief in certainty of knowledge rated these questions as more answerable, *r*(226) = 0.28, *p* < 0.001. Also, people who believed more in mankind’s efficacy rated the non-consensus questions as more answerable, *r*(216) = 0.22, *p* = 0.001.

In order to explore how much variance in the answerability ratings of the non-consensus questions was explained by the individual difference variables when the current answerability scale was used, a multiple regression with the dependent variable answerability of non-consensus questions and the independent variables belief in certainty of knowledge, EPI-Default, EPI-Intellectual, mankind’s efficacy, optimism and mankind’s knowledge was conducted. The result was significant *F*(4,209) = 5.26, *p* < 0.001. The regression analysis showed that the individual difference variables explained 13% of the variance in the answerability judgments. Furthermore, belief in certainty of knowledge (beta value = 0.23), mankind’s efficacy (beta value = 0.13) and optimism (beta value = -0.14) yielded significant β-values.

#### Illusion Questions

A negative correlation was found between the answerability ratings of the illusion questions and optimism, thus, the more optimistic people rated illusion questions as less answerable, *r*(216) = -014, *p* < 0.047.

One hundred and one participants in the current scale condition rated both of the illusion questions as 100% answerable, and were thus “tricked” by the illusion questions. Interestingly, on average these 101 participants also rated each of the other questions higher or equally high in answerability compared to other participants. In order to investigate if epistemic processing preference differed between the tricked and non-tricked participants, a general EPI-score was computed with the formula (EPI-score = 5 + EPI-default – EPI-intellectual, where a higher score means a higher preference for default processing). Tricked participants did not score significantly higher on the EPI–score.

### Individual Difference Variables and Future Answerability

The correlations between the future answerability scale and the individual difference measures can be found in **Table 3.**

**Table 3:** Spearman’s rho correlations between the individual difference variables and the answerability ratings of the consensus, non-consensus, and illusion questions on the future answerability scale.

	1	2	3	4	5	6	7	8	9
(1) Belief in certainty of knowledge		0.30^∗∗^	-0.04	0.32^∗∗^	0.18^∗∗^	-0.05	0.11	-0.10	0.07
(2) EPI-default			-0.23^∗∗^	0.16^∗∗^	0.21^∗∗^	0.02	0.09	-0.03	-0.13
(3) EPI-intellectual				0.11^∗^	-0.06	-0.06	0.01	-0.01	0.19^∗∗^
(4) Mankind’s efficacy					0.15^∗∗^	0.21^∗∗^	0.05	-0.15^∗^	-0.01
(5) Mankind’s knowledge						0.04	0.11	-0.15^∗^	-0.14^∗^
(6) Optimism							-0.02	0.00	-0.05
(7) Consensus								0.10	0.10
(8) Non-consensus									0.06
(9) Illusion									


*^∗^*p* < 0.05, ^∗∗^*p* < 0.01.*

#### Consensus and Non-Consensus Questions

No significant correlations (*p* < 0.05) were found between the consensus questions and the individual difference measures for the future answerability scale. As can be seen in **Table 3**, rating the non-consensus questions to be more answerable in the near future was associated with higher ratings for mankind’s efficacy *r*_s_(214) = -0.15, *p* = 0.028, and mankind’s knowledge *r*_s_(214) = -0.15, *p* = 0.026.

The correlation between mankind’s knowledge and the answerability values for the non-consensus questions was stronger when belief in certainty of knowledge was rated first, *r*_s_(113) = -0.27, *p* = 0.004. Likewise, the correlation between mankind’s efficacy and the answerability values for the non-consensus questions was more pronounced when belief in certainty of knowledge was judged before answerability judgments, *r_s_*(113) = -0.23, *p* = 0.016.

#### Illusion Questions

As can be seen in **Table 3**, rating the illusion questions as being more answerable in the near future was associated with higher ratings for mankind’s knowledge *r*_s_(214) = -0.14, *p* = 0.036, and lower EPI-intellectual rating, *r*_s_(214) = 0.19, *p* = 0.005. Thus, higher answerability ratings for the illusion questions were associated with lower values on the EPI-intellectual scale.

In total 182 participants claimed the illusion questions could be answered today and were thus tricked by the illusion questions. Comparing the medians, tricked participants judged each of the other questions to be answered at the same time or sooner than non-tricked participants. The tricked participants (*n* = 182) had a significantly lower EPI-score (computed with the formula EPI-score = 5 + EPI-default – EPI-intellectual, where a higher score means a higher preference for default processing), *t*(432) = -2.20, *p* = 0.028, *d* = -2.20.

## General Discussion

This study investigated judgments of current and future answerability of three types of knowledge questions: consensus questions (high level of expected consensus about their answerability), non-consensus questions (lower expected level of consensus about their answerability), and illusion questions (computational questions with crucial information lacking). We also investigated whether there was a relationship between various individual difference variables and the level of the answerability judgments.

Questions rated low on answerability on the current scale were also rated to be answerable further off in the future. The finding that the level of judged answerability was fairly strongly correlated between the two scales indicates that there is some stability in the processes generating answerability judgments. This result is also in line with the conclusion reached by Hampton et al. (2012) that people in the context of general knowledge statements can show agreement on what are known unknowns. However, as discussed below, some differences were found between the two scales with respect to their sensitivity for the participants’ thought activity prior to making the answerability judgment.

The consensus questions were rated as having a high level of answerability and were, as expected, judged as more answerable than the non-consensus questions, independent of the scale used. A possible explanation for the high ratings of the consensus questions is that fairly automated memory processes more often quickly identified a correct answer or classified the consensus questions as being a type of question that could be known by myself or by others. This explanation coheres with research on “don’t know” judgments which shows that spontaneous quick processes may quickly contribute to the assessment of many questions (e.g., Glucksberg and McCloskey, 1981). Results that also supported the importance of quick automatic processes when answering questions were reported by Reder and Ritter (1992). These researchers found that people, when answering a question, first evaluate the question by using a type of feeling of knowing judgment based on the features of the questions asked. Our suggested explanation is also in line with Koriat’s (2012) finding that experienced consensus about an answer was associated with increased confidence that the answer was correct.

The non-consensus questions were given the lowest answerability ratings of the three question types. In line with the considerations above, the answerability ratings for these questions may have been more dependent on elaborated and deliberative thinking, compared to the consensus questions. For example, it is likely that there was less experienced consensus about a possible answer to many of these questions and that for this reason the advice offered from socially prevalent knowledge (compare Koriat, 2008, 2012; Shtulman, 2013) may have been less obvious or clear, thus increasing the possibility that the participants will engage in more independent deliberative thinking. The possibility that the answerability judgments for the consensus and non-consensus questions may have been influenced by somewhat different processes was also indicated by the finding, further discussed below, that the individual difference variables were mostly associated with the non-consensus questions.

Many participants rated the illusion questions as answerable today even though crucial information was missing. The results for the illusion questions indicate that a “feeling of others knowing” can be created even when it is unlikely that somebody can come up with an answer. The participants who were tricked by the illusion questions showed tendencies to have an epistemic preference for higher default processing in combination with lower intellectual processing. High answerability judgments for the illusion questions may therefore possibly be explained by shallow processing based on general beliefs (Bromme et al., 2010), in contrast to deeper processing of the actual question item *per se*. Interestingly, participants who failed to notice the missing information in the illusion questions also rated other questions either higher or equally high in answerability.

### Individual Difference Measures and the Answerability Judgments

Overall, the individual difference variables all showed acceptable or satisfactory Cronbach’s alpha values and were moderately related to answerability judgments. In line with our hypothesis, when an effect was found it was related to the answerability of non-consensus questions, not to the consensus questions. However, it should be noted that the weaker correlations for the consensus questions may also be due to their lower variability, compared with that of the non-consensus questions and this issue should be further explored in future research.

Together the individual difference variables explained 13% of the variance of the answerability judgments for non-consensus questions when the current scale was used. This can be seen as an irrelevant influence on the level of the answerability judgments in the sense that variance in individual difference variables is not usually considered likely to contribute to better quality in judgments, that is, more realistic values. Future research should investigate if there are specific levels of some individual difference variables that are beneficial for producing realistic answerability judgments. Generally speaking, in important applied contexts it may be worthwhile to develop approaches that can help to decrease the negative influence of individual difference variables.

It is also noteworthy that the participants who believed more in certainty of knowledge also rated the more challenging questions (the non-consensus questions) as more answerable, especially for the current scale, providing some support for our hypothesis. They also tended to believe more in mankind’s knowledge (that more is known of all there is to know and that is important to know) and especially to believe more in mankind’s efficacy with respect to solving difficult problems and troubles. These associations may also have contributed to the tendency for participants who believed more in mankind’s knowledge and efficacy to give higher answerability ratings. Future research should investigate whether people who believe more in certainty of knowledge and mankind’s efficacy differ from other people in the realism of their answerability judgments. For example, do such people, when they consider real-life complex issues, have a tendency to underestimate the number of variables relevant to consider or the complexity of the possible interactions between variables?

When it comes to epistemic preferences, we expected that preference for default processing would be associated with higher answerability judgments and intellectual processing with lower answerability judgments. Although the earlier mentioned result showed that participants high in default processing and low in intellectual processing were tricked more by the illusion questions, only a weak correlation was found between intellectual processing and low answerability for the illusion questions when using the future scale. Although it was small, this correlation supports our hypothesis. Since people high in intellectual processing enjoy and take time to think about problems (Elphinstone et al., 2014), it is likely that these individuals identified that the illusion questions lacked a component to be answerable.

The results for the relation between personal optimism and answerability generally showed quite weak correlations which became significant or non-significant depending on method of analysis. Optimism was also significantly, but weakly, correlated with *lower* answerability ratings on the non-consensus and the illusion questions for the current answerability scale. According to Peterson (2000) personal optimism is characterized by either *explanatory style* or *dispositional* optimism. Explanatory style relates to how the individual tends to explain good or bad outcomes and may be less relevant to the present study. Dispositional optimism means a tendency to expect good things and few bad things to happen in the future, to believe that one can reach one’s goals (e.g., optimism correlated between *r* = 0.2–0.6 with self-efficacy in different countries in the study by Luszczynska et al., 2005), to make more attempts to reach one’s goals and to not give up. Similarly, research has found optimism to correlate with overconfidence (Wolfe and Grosch, 1990). These features would seem to indicate that optimists would show a tendency to give higher answerability ratings. However, optimists can also be expected to be able to recognize and tolerate higher degrees of uncertainty since they expect outcomes to be good. In line with this, Scheier et al. (1986) found optimists to be associated with less denial/distancing. Since the tendency in the correlations both for the non-consensus and the illusion questions was that optimists tended to give low answerability ratings, this later tendency showed to be more important than the first listed features of personal optimism for determining the level of the answerability judgments. However, the empirical evidence for this conclusion should be replicated with new samples and questions in future research.

As already noted, the results showed that answerability judgments of the consensus questions were not appreciably influenced by the individual difference variables. This could be due to a ceiling effect since the consensus questions received very high ratings, limiting the spread of their answerability ratings. However, the fact that the answerability ratings for the two types of questions were significantly correlated (see **Table 2**) speaks against this interpretation, although this correlation, in spite of it being significant, was relatively low. It would seem more likely that the lack of associations between the consensus questions and the individual difference variables is a true effect in the sense that individual difference variables, for reasons discussed above, may have had less influence on the judgments of these questions, although future research should examine this issue further.

Interestingly, rating belief in certainty of knowledge before judging the answerability questions on the future answerability scale made participants think the non-consensus questions were more answerable. The correlations between mankind’s knowledge and mankind’s efficacy and answerability of the non-consensus questions were also more pronounced when the ratings about belief in certainty of knowledge were made before answering the answerability questions. Given the general activating effect of priming in memory (Tulving and Schacter, 1990), one can speculate that making ratings about certainty of knowledge by evaluating positively formulated statements about the possibility to find the truth, as was the case in the present study, may have influenced the information activated in the participants’ memory. For example, it may have made the participants who rated their belief in certainty of knowledge before the answerability ratings attend more to all the things mankind knows and can do, taking focus away from human knowledge flaws and this may have contributed to higher answerability ratings.

### Limitations and Further Research

The present study represents an early attempt to investigate some aspects of question answerability judgments and has various limitations. The results showed that our participants had a fairly sanguine view of the answerability of the questions we tested, as most of the questions were seen as answerable today or in the future. Most of the questions’ answerability was considered just a matter of time, and not beyond human reach. However, the participants in our study (being members of our participant pool) can be argued to have a fairly positive attitude toward research and science. Future research should test if our results generalize to more representative groups of people and to people faced with uncertain but important questions, for example, researchers and politicians. Furthermore, future research should preferably use a larger number of answerability questions, especially a larger number of illusion questions.

Finally, future research should also investigate people’s answerability judgments of other types of questions than in the present study. Examples are questions relating to current controversies in science and questions more clearly related to social life, such as questions whether a suspect can be considered guilty “beyond reasonable doubt” (see e.g., Dhami, 2008), questions concerning the effects of previous or planned social reforms in different societal contexts, and questions relating to scientific issues and supernatural issues.

## Author Contributions

BK has with CMA had the main responsibility for data-collection, analysis, conclusions and creation of the manuscript. BK and SB have performed approximately half of the statistical analyses each. Furthermore, SB has actively supported the development of the study and has several times reviewed the paper and contributed to the Introduction and the Discussion with formulations and interpretations of theories.

## Conflict of Interest Statement

The authors declare that the research was conducted in the absence of any commercial or financial relationships that could be construed as a potential conflict of interest.

## Footnotes

1 Nine further participants were excluded since they in response to a manipulation check question answered before the questions on the individual difference variables were answered, understood the current answerability scale as “100% means that 100% of the population can answer the question.” This interpretation was identified in pre-studies by means of a manipulation check multi-alternative question after the answerability questions. This interpretation could be a misunderstanding, but it could also serve as a (limited) legitimate strategy for judging question answerability.

2 In order to ensure the robustness of the ANCOVA, non-parametric analysis was also conducted (Friedman, Wilcoxon). Since the result was in line with the ANCOVA we report only the ANCOVA.

## Appendix 1

### Questions

The questions were presented in following domains: Swedish grammar, Space, Geometry, South-American history, Galaxies, Physics, Medicine, Diseases, Climate science, Tics, and Technology. Three categories of questions were used: consensus, non-consensus, and consensus illusion. Respondents were asked to make judgment of answerability either on a current answerability scale or a future answerability scale. A complete list of the questions with question category in parenthesis follows below.

### Consensus Questions

• What word-class does the word beautiful belong to? (Swedish grammar)
• What is the name of our galaxy? (Galaxies)
• What is a super-nova? (Space)
• The formula for a circumference of a circle is denoted π∗D. What does D stand for? (Geometry)
• How large is the voltage if the electric current is 10 amp and the resistance 15 k? (Physics)
• The virus that causes chicken pox can also cause another illness. Which one? (Diseases)
• What is the branch of medicine that deals with the uses, effects, and modes of drugs called with another word? (Medicine)
• What century did Columbus start the European colonization of South America? (South-American history)

### Non-Consensus Questions

• How many varieties of the word “snow” did humans have during the ice age? (Swedish grammar)
• How many galaxies are there in the universe? (Galaxies)
• What proportion of all diseases has psychological causes? (Diseases)
• How many varieties of bacteria can be transmitted to humans via tics? (Tics)
• How much environmentally hazardous material does the anno 2014 most common laptop contain? (Technology)
• Are humans causing global warming? (Climate science)
• Will the polar ices melt in before year 2514? (Climate science)
• Is there life in space? (Space)
• Is there anno 2014 a safe technology for storing nuclear waste? (Technology)
• Does the vaccination used year 2014 provide a safe protection against the disease TBE, submitted via tics? (Tics)
• Does the human body anno 2014 have a so far unknown circulation system? (Medicine)
• Did the Maya Indians have any knowledge about the end of the world? (South-American history).

### Illusion Questions

• How large is the area of an ellipse with a minor axis of 2 cm? (Geometry)
• How large is the electric effect at a voltage of 100 V? (Physics)

The standard deviation reflects the variability of each response for each individual across the sample of individuals. *Consensus* questions are marked with “C”, *Non-consensus* questions with “NC”, and *Illusion* questions with “I”.

## Appendix 2

### Instructions to Current Answerability Judgments

For each question we would like you to answer how probable it is that the question *is possible to answer*. We are thus not asking for the particular answer, but what you believe about the *answerability of the question*. In order to make the question count as 100% answerable it is enough that at least one now living person in the whole world can answer the question. Zero percent means that nobody can answer the question. You can also choose a probability between 0 and 100%, e.g., the probability that the question is answerable is 90%. With 100% we mean that the question can be answered in a correct way and that you can provide good arguments for the answer.

### Instructions to Future Answerability Judgments

For each question we would like you to answer *if the question is possible to answer* and in that case when. We are thus not asking for the particular answer but what you think about *the question’s answerability*. That is, is there anyone who can answer the question today, in the future, or will the question never be answered?

In order to make the question count as answerable it is enough that at least *oneperson* in the whole world can answer the question. With answerability we mean that the question can be answered in a *correct* way and that you can provide *good argument* for the answer. Answer options will be presented on a timescale…the alternatives on the time-scale are mutually exclusive. That is, “within 1 year” means 0–1 year, “within 10 years” means 2–10 years, and “within 20 years” means 11–20 years, etc…
